# Modulation of Potassium Channels Inhibits Bunyavirus Infection*

**DOI:** 10.1074/jbc.M115.692673

**Published:** 2015-12-16

**Authors:** Samantha Hover, Barnabas King, Bradley Hall, Eleni-Anna Loundras, Hussah Taqi, Janet Daly, Mark Dallas, Chris Peers, Esther Schnettler, Clive McKimmie, Alain Kohl, John N. Barr, Jamel Mankouri

**Affiliations:** From the ‡ School of Molecular and Cellular Biology, University of Leeds, Leeds LS2 9JT,; § School of Life Sciences, University of Nottingham, Nottingham NG7 2UH,; ¶ Veterinary Medicine and Science, University of Nottingham, Nottingham NG7 2RD,; ‖ School of Pharmacy, University of Reading, Reading RG6 6AP, and; ** MRC-University of Glasgow Centre for Virus Research, Scotland, Glasgow G61 1QH, United Kingdom

**Keywords:** antiviral agent, ion channel, negative-strand RNA virus, pathogenesis, potassium channel

## Abstract

Bunyaviruses are considered to be emerging pathogens facilitated by the segmented nature of their genome that allows reassortment between different species to generate novel viruses with altered pathogenicity. Bunyaviruses are transmitted via a diverse range of arthropod vectors, as well as rodents, and have established a global disease range with massive importance in healthcare, animal welfare, and economics. There are no vaccines or anti-viral therapies available to treat human bunyavirus infections and so development of new anti-viral strategies is urgently required. Bunyamwera virus (BUNV; genus *Orthobunyavirus*) is the model bunyavirus, sharing aspects of its molecular and cellular biology with all *Bunyaviridae* family members. Here, we show for the first time that BUNV activates and requires cellular potassium (K^+^) channels to infect cells. Time of addition assays using K^+^ channel modulating agents demonstrated that K^+^ channel function is critical to events shortly after virus entry but prior to viral RNA synthesis/replication. A similar K^+^ channel dependence was identified for other bunyaviruses namely Schmallenberg virus (*Orthobunyavirus*) as well as the more distantly related Hazara virus (*Nairovirus*). Using a rational pharmacological screening regimen, two-pore domain K^+^ channels (K_2P_) were identified as the K^+^ channel family mediating BUNV K^+^ channel dependence. As several K_2P_ channel modulators are currently in clinical use, our work suggests they may represent a new and safe drug class for the treatment of potentially lethal bunyavirus disease.

## Introduction

The *Bunyaviridae* family represents the largest taxonomic grouping of negative sense RNA− viruses, with over 350 named members (1). The family is divided into five genera, *Orthobunyavirus*, *Hantavirus*, *Tospovirus*, *Phlebovirus*, and *Nairovirus* and takes its name from Bunyamwera virus (BUNV),^3^ the prototype of the genus *Orthobunyavirus*.

All bunyaviruses share common elements of virion structure being enveloped and containing an RNA genome that comprises three separate RNA segments named small (S), medium (M), and large (L). These three segments encode four structural proteins using an expression strategy that is conserved across all members of the family: the S segment encodes the nucleoprotein (N), the M segment encodes two glycoproteins (Gn and Gc), and the L segment encodes an RNA-dependent RNA polymerase (L protein). Most *Bunyaviridae* family members including BUNV also encode two non-structural proteins; NSs from the S segment, and NSm from the M segment (2, 3).

Bunyaviruses are predominantly arthropod-borne viruses capable of infecting a wide range of hosts including humans, plants, and animals. Four of the five bunyavirus genera include members that are associated with lethal hemorrhagic fevers in infected humans, and many are considered to be emerging pathogens due to a complex combination of factors including travel, animal trade, and climate change (4–6). Bunyavirus emergence is facilitated by the segmented nature of their genome that allows reassortment between different species to generate novel viruses with altered pathogenicity (7). Schmallenberg (SBV) and Ngari (NGAV) viruses are orthobunyaviruses that exemplify this phenomenon; NGAV, which causes a highly fatal hemorrhagic fever in humans (8) is a reassortant possessing S and L segments from BUNV and the M segment from the closely related Batai virus (BATV) (9). Similarly, SBV, which is a pathogen responsible for teratogenic disease in sheep and cattle (10), has a complex genetic background possessing segments shared with previously characterized Sathuperi and Shamonda viruses (11). The threat of widespread arthropod borne transmission of these potentially lethal emerging viruses means the development of preventative and therapeutic strategies is urgently required.

One strategy to identify new anti-viral therapies is to target virus host interactions that are essential for virus multiplication. Many examples of such interactions have been described for bunyaviruses (reviewed in Refs. 12, 13) and other recent examples include those involved in virus entry (14), counteracting the antiviral response (15–17) and modulating host gene expression (16, 18). Another group of potential targets are host cell ion channels, which regulate ion homeostasis across all cellular membranes and are key players in a broad and extensive range of cellular processes including the cell cycle, gene expression, cell signaling, and innate immunity (19–21). Ion channels are emerging as key factors required during virus replicative cycles, and have been assigned critical roles in virus entry, survival, and release. Examples include roles for both potassium (K^+^) and chloride ion (Cl^−^) channels during the hepatitis C virus lifecycle (22, 23), the ability of human immunodeficiency virus proteins to modulate K^+^ channel activity (24, 25) and the recently identified requirement of two-pore calcium (Ca^2+^) channels (TPCs) in endosomal membranes for successful entry of Ebola virus (26). Here, we report that BUNV multiplication depends on the function of cellular K^+^ channels. Using the available ion channel pharmacological tools, a small family of two-pore potassium ion channels (K_2P_) was identified as the candidate K^+^ channel family critical for BUNV multiplication. We propose that targeting K^+^ channel function may represent a new, pharmacologically safe and broad ranging therapeutic strategy for this important family of pathogens.

## Experimental Procedures

### 

#### 

##### Cells and Viruses

A549 (adenocarcinomic human alveolar basal epithelial cells) and Vero (African green monkey kidney cell lines) cells were obtained from the European Collection of Cell Cultures (ECACC) and maintained at 37 °C in Dulbecco's modified Eagle's medium (DMEM-Sigma) supplemented with 10% fetal bovine serum (FBS), 100 units/ml penicillin and 100 μg/ml streptomycin. Huh7 cells (hepatocyte derived cellular carcinoma cell line) were a gift from R. Bartenschlager (Heidelberg University) and maintained under these conditions but additionally supplemented with non-essential amino acids. BSRT-7 cells (derived from BHK21 cells, that constitutively express bacteriophage T7 polymerase) were obtained from K. K. Conzelmann (University of Munich) (27) and maintained under these conditions but additionally supplemented with G418 (100 μg/ml). U87-MG cells (a human glioblastoma-astrocytoma, epithelial-like cell line) were obtained from the ECACC and grown in minimal essential Eagle's medium (MEM) supplemented with 10% FBS, 1 mm sodium pyruvate, 1 mm non-essential amino acids, 2 mm l-glutamine, penicillin G (100 units/ml), and streptomycin (100 μg/ml). All mammalian cells were incubated in a humidified incubator at 37 °C in the presence of 5% CO_2_. *Ae. albopictus* C6/36 cells (a gift from L. Alphey, University of Oxford) were grown at 28 °C in Leibovitz's 15 medium (Sigma) supplemented with 10% FBS and 10% tryptose phosphate broth. Working stocks of wild-type BUNV (provided by R. Elliot, Glasgow Centre for Virus Research) was prepared as described previously (28). HAZV (strain JC280, ECACC) was propagated in SW13 cells (human adrenal cortex carcinoma cell line, ECACC) and clarified supernatants were used to infect A549 cells. The titer of infectious HAZV was estimated by plaque assay; typical yields from virus propagation experiments were between 1 × 10^5^ and 1 × 10^7^ plaque forming units per ml (pfu/ml). Human RSV strain A2 (subgroup A, ECACC) was passaged through HEp-2 cells (human cervix carcinoma cell line, ECACC) and purified by sucrose density gradient according to previously described protocols (29). Viral titers were accurately determined in HEp-2 cells by a modified methylcellulose-based plaque assay with immunostaining using goat anti-hRSV antibodies (Abcam). SBV (strain BH80/11-4 provided by M. Beer, Friedrich-Loeffler Institute) was propagated in Vero cells in the presence of 2% FBS.

##### Drug Treatments

For the assessment of BUNV infection, 1 × 10^5^ cells/well were seeded into 6-well plates and allowed 24 h to settle. Where indicated, cells were pre-incubated with the specific ion channel modulator for 45 min, or medium supplemented with the solvent control of each channel modulator, prior to and during virus infection. Cells were infected at an MOI of 1 for 24 h.

##### Assessment of Virus Production by Western Blot Analysis

BUNV-N protein expression was assessed using the following method. Infected cells were lysed in GLB buffer (25 mm glycerol phosphate, 20 mm Tris, 150 mm NaCl, 1 mm EDTA, 1% Triton X-100, 10% glycerol, 50 mm NaF) plus protease inhibitors (Complete; Roche). Cell lysates (50 μg of protein) were normalized by BCA assay and resolved by SDS-PAGE, transferred to a PVDF membrane (Millipore) using a Bio-Rad Laboratories semidry transfer apparatus, and probed with a sheep BUNV-N serum followed by labeling with anti-sheep HRP-conjugated secondary antibodies (Sigma). All Western blots were visualized using ECL system (Advansta). HAZV-N expression was assessed using a sheep HAZV-N serum before labeling with anti-sheep HRP conjugated secondary antibodies (Sigma). For HRSV staining, cells were labeled with goat anti-hRSV antibodies (Abcam) and anti-goat HRP-conjugated secondary antibodies (Pierce).

##### Immunofluorescent Analysis

For the assessment of BUNV infection, infected cells grown on polylysine-coated glass coverslips were fixed with methanol for 10 min, permeabilized in ice-cold methanol/acetone for 10 min, and blocked in PBS/1% BSA for 30 min. For BUNV-N protein staining, cells were labeled with sheep anti-BUNV-N serum before staining with sheep Alexa Fluor 594 nm conjugated anti-sheep secondary antibodies (Invitrogen-Molecular Probes) in PBS/1% BSA. Cells were washed and mounted onto microscope slides using prolong gold containing DAPI (Invitrogen-Molecular Probes). Labeled cells were viewed on an inverted Zeiss 510-META laser-scanning confocal microscope under an oil-immersion 40 or 63× objective lens (NA = 1.40). Alexa Fluor 594 nm (550 nm excitation, 570 nm emission) was excited using a helium/neon laser fitted with 543 nm filters. Displayed images are representative of at least three independent experiments and displayed as single optical sections of 50 μm thickness. Randomly chosen fields were observed under fluorescence microscopy to assess virus staining and the number of virus-positive cells quantitated for ≥100 cells per condition using ImageJ software as previously described (30). Results are presented as means ± S.E. from a minimum of three independent experiments. For HAZV-N staining, cells were labeled with sheep anti-HAZV-N serum before staining with sheep Alexa Fluor 594 nm conjugated anti-sheep secondary antibodies. For hRSV staining cells were labeled with goat anti-hRSV antibodies (Abcam) and stained with goat Alexa Fluor 594 antibodies (Invitrogen-Molecular Probes). For the assessment of endogenous K_2P_ channel expression, fixed A549 cells were labeled with anti-KCNK 1, 2, 7, or 9 antibodies (Santa Cruz Biotechnology Inc) and stained with the appropriate Alexa Fluor 488/594 antibodies (Invitrogen-Molecular Probes).

##### Time-course Assays

BUNV infected cells were treated with TEA (10 mm) or quinidine (200 μm) in the medium during the 24 h infection period (T = 0), or added 1, 2, 4, 6, 8, and 10 hpi. Virus infection was allowed to proceed for a total of 24 h, and cells were lysed for Western blot analysis. For the assessment of BUNV entry, A549 cells were treated during the initial 1h of BUNV infection with TEA (10 mm) or monensin (20 μm). BUNV virions that were not internalized were washed off with 0.5% trypsin in PBS (×3). Medium was then replaced, and the cells incubated for 24 h before analysis.

##### BUNV Growth Assays

BUNV was propagated in Vero cells in DMEM containing 2% FBS. Culture supernatants were harvested when >90% cytopathic effect (CPE) was observed and passed through a 0.45 μm syringe filter to removed cellular debris. For virus inhibition experiments, growth medium was removed from Vero cells and replaced with 2% FBS DMEM, supplemented with the appropriate drug dilution, for 1 h at 37 °C. Medium was transferred to empty plates while cells were incubated for 1 h at 37 °C with serum-free medium (Mock) or serum-free medium containing 500 TCID_50_/ml BUNV (MOI ∼0.025). Medium was discarded and drug containing-medium was returned, and cells incubated for 5 days. Cells were washed and fixed in 100% ethanol for 30 min at room temperature before staining with 3% (*w*/*v*) crystal violet for 1 h at room temperature.

##### BUNV Transcription/Replication

A549 cells were infected with BUNV (MOI 1) and either treated with TEA (10 mm), or left untreated. At 1, 6, or 24 hpi, total cell RNA were harvested using an RNeasy kit (Qiagen). RNA from mock-infected A549 cells was also harvested. All RNAs were subjected to primer extension analysis as previously described (31), using ^33^P end-labeled oligonucleotide primer P-ext (5′-GAGCCTTTAATGACCTTCTGTTGG-3′) designed to anneal to nucleotides 58–81 of the BUNV S segment anti-genome. Extension products were separated by 6% denaturing PAGE, and visualized using autoradiography. BUNV replicon assays were performed as previously described (12, 31). Briefly, BSRT7 cells were transfected with cDNA plasmids encoding BUNV S and L segment open reading frames (ORFs), and a model negative sense RNA segment that contained conserved BUNV 3′ and 5′ non-translated regions flanking the *Renilla* luciferase ORF. Cells were incubated with or without TEA (5 or 10 mm) for 18 h and lysed in passive lysis buffer (Roche). Cell lysates were analyzed for luciferase activity (relative luminescence units (RLU) per second) as a measure of BUNV gene expression. The L ORF plasmid was omitted and replaced with GFP to act as a negative control to measure non-BUNV mediated luciferase expression. Results were normalized to those for an untreated control. Error bars represent the standard errors of the means (S.E.) of results from three independent experiments performed in duplicate.

##### Resting Membrane Potential Assay

Cells were treated with 20 μm DiBAC4(3) (Sigma) in medium for 20 min at 37 °C and protected from light. Conditions were selected as optimal from standardization of DiBAC4(3) concentrations, incubation time, and temperature. Following labeling, cells were washed and either mock-infected or infected with BUNV (MOI 1). Cells were treated with TEA (10 mm) or quinidine (200 μm) as controls. The average mean intensity of green fluorescence GCU (Green Calibration Units per well) was measured from several widefield images using IncuCyte ZOOM live cell imaging at 1-h intervals for up to 6 h. Values are presented as the mean fluorescent intensity per cell normalized to untreated controls from a minimum of three independent experiments. Assays of [Cl^−^]*_i_* using 5 mm 6-methoxy-quinolyl acetoethyl ester (MQAE) were performed as previously described (22). 5-Nitro-1–3-phenylpropylamino benzoic acid (NPPB) and indyanyloxyacetic acid 94 (IAA-94) were included as compounds known to modulate Cl^−^ function for assay verification.

##### K_2P_ Channel RT-PCR

Total cell RNA was extracted from A549 cells and reverse transcribed under the following conditions: 3 min 94 °C; 30 cycles, 45 s 94 °C, 30 s 55 °C, and 90 s 72 °C; final extension 10 min 72 °C. Primers for KCNK1–17 are available on request. PCR products were resolved on 2% agarose gels.

##### Statistical Analyses

The statistical significance of data were determined by performing a Student's *t* test or one way ANOVA test. Significance was deemed when the values were less than or equal to the 0.05 *p* value.

## Results

### 

#### 

##### K^+^ Channels Are Required for BUNV Infection

**FIGURE 1.**
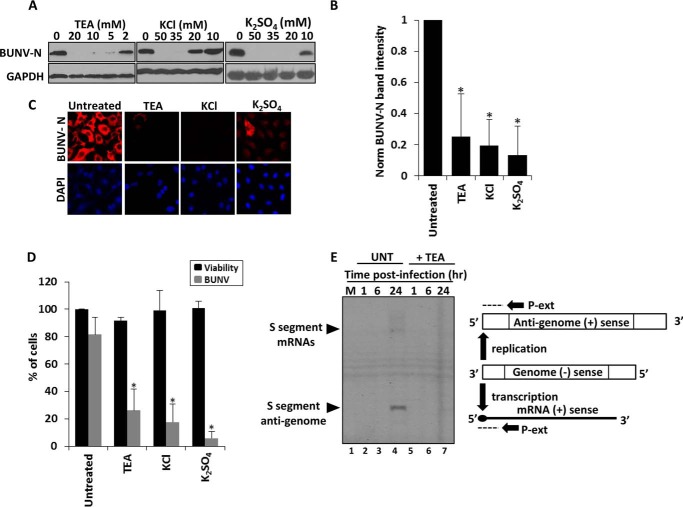
**K^+^ channel inhibition impedes BUNV infection.** *A*, representative Western blots from BUNV-infected A549 cells (24 h, MOI 1) treated with the indicated K^+^ (TEA, KCl, K_2_S0_4_), channel blockers. Lysates were resolved by SDS-PAGE and probed with sheep anti-BUNV N serum (*n* ≥ 3). Blots were reprobed for GAPDH as a loading control. *B*, densitometry of Western blots from Fig. 1*A* from TEA (10 mm), KCl (40 mm), K_2_S0_4_ (40 mm), performed using ImageJ. Signals were normalized to BUNV infected, no-drug controls (*n* = 3). Results are expressed as mean ± S.E. (*, significant difference at the *p* ≤ 0.05 level; *NS*, not significant. *C*, cells plated onto coverslips were treated with TEA (10 mm), KCl (20 mm), K_2_S0_4_ (20 mm), and BUNV infected for 24 h. Cells were fixed, permeabilized, and stained for BUNV-N (*red*) and DAPI (*blue*) using sheep anti-BUNV-N serum and sheep Alexa-fluor 594 antibodies. *D*, % of BUNV-infected cells was assessed for each treatment from widefield images of BUNV-N stained cells (*gray bars*). A minimum of 100 cells were counted. Results are expressed as mean ± S.E. (*, significant difference at the *p* ≤ 0.05 level; *NS*, not significant). A549 cells were treated as above and cell viability assessed via ATPlite assays (*black bars*). Values are normalized to untreated controls. *E*, A549 cells were infected ± 10 mm TEA and RNA were extracted at the indicated time points and subjected to primer extension analysis to quantify S segment anti-genomes and mRNAs using ^33^P end-labeled primer P-ext. The position at which P-ext annealed to BUNV specific positive sense RNAs is shown schematically. Resulting extension products were analyzed by denaturing 6% PAGE and visualized by autoradiography.

To determine if the activity of host cell K^+^ channels are required during the bunyavirus lifecycle, BUNV infection assays were performed in the presence of broad-spectrum K^+^ channel modulators used at pharmacologically relevant concentrations. BUNV growth was assessed through detecting the expression of the BUNV nucleoprotein (BUNV-N); known to correlate with virus production (28). Tetraethylammonium (TEA), a broad spectrum K^+^ channel blocker, inhibited BUNV infection at concentrations ≥2 mm (Fig. 1, *A* and *B*). A similar effect was seen when extracellular KCl was increased above 20 mm (Fig. 1, *A* and *B*) to collapse K^+^ gradients and inhibit K^+^ channel activity. Any non-K^+^ channel effects due to the off target effects of these compounds were ruled out as a second K^+^ salt K_2_S0_4_ similarly inhibited BUNV-N production at concentrations ≥20 mm (32–37). These data were confirmed when the percentage of BUNV-infected cells was assessed by analysis of immunofluorescence staining for BUNV-N (TEA = 73.85%± 15.3 reduction *p* ≤ 0.05, KCl = 82.32%± 13.2 reduction *p* ≤ 0.05, K_2_S0_4_ = 94.20%± 5.0 reduction *p* ≤ 0.05), (Fig. 1, *B* and *C*, *gray bars*). At the concentrations assessed, all compounds were non-toxic as confirmed by ATPlite^TM^ viability assays (Fig. 1*D* (*ii*) *black bars*). To further confirm the findings, the effects of K^+^ channel inhibition were assessed on the accumulated RNA products of either BUNV mRNA transcription or genome replication using primer extension (Fig. 1*E*). At the 24 h time point, extension products corresponding to BUNV mRNA transcripts and BUNV anti-genomic RNAs were detectable in BUNV-infected cells. In contrast, extension products were undetectable in BUNV-infected cells treated with TEA (Fig. 1*E*, compare *lanes 4* and *7*). Taken together, these data indicate a specific requirement for K^+^ channel function during the BUNV replicative cycle.

##### K^+^ Channel Dependence Is Conserved Across Invertebrate and Vertebrate Cell Types

**FIGURE 2.**
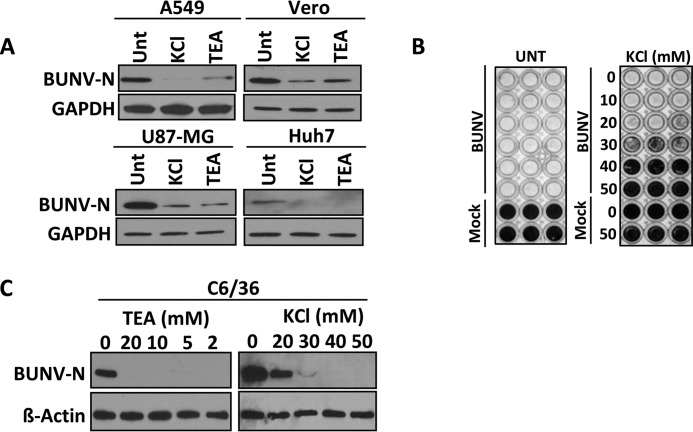
**K^+^ channel dependence is conserved across invertebrate and vertebrate cell types.** *A*, indicated mammalian cell lines were treated with 40 mm KCl or 10 mm TEA and infected with BUNV (24 h, MOI 1). BUNV-N production was assessed as in Fig. 1*A. B*, Vero cells were infected with 500 TCID_50_/ml BUNV in the presence of KCl or TEA for 5 days. Cells were fixed in 100% ethanol and stained with 3% (*w*/*v*) crystal violet. Representative plates of three independent experiments are shown. *C*, C6/36 *Ae. albopictus* cells were treated with the indicated concentrations of TEA and KCl and infected with BUNV in an identical procedure to *A*. Blots were reprobed for β-actin as a loading control.

As expected from its arthropod-borne transmission to a variety of mammalian hosts, BUNV can replicate in a range of vertebrate and invertebrate cell lines (3, 4). The dependence of BUNV on K^+^ channel function was therefore assessed in a range of cell types. Vero (monkey kidney cells) U87-MG (human glioblastoma cells), Huh7 (human hepatocellular carcinoma cells) supported BUNV infection, as evidenced by robust BUNV-N expression (Fig. 2*A*), which was inhibited by the addition of TEA and KCl. To rule out the possibility of the observed inhibition being restricted to BUNV-N expression, the effects of K^+^ channel inhibition on virus infection and spread was assessed. BUNV induces extensive cytopathic effect (CPE) in Vero cells resulting in the formation of plaques under solid culture or the destruction of the monolayer under liquid culture. In untreated Vero cells infected with BUNV at a low MOI (∼0.025), the cytopathic effect (CPE) was so extensive after 5 days of culture that no cell monolayer was present to take up stain (Fig. 2*B*). In contrast, treatment of BUNV-infected cells with ≥30 mm KCl abrogated virus-induced CPE, confirming that K^+^ channel modulation inhibits BUNV production. Numerous K^+^ channels are expressed in insect cells (38, 39). Indeed *Drosophila* ATP-sensitive K^+^ channels have been demonstrated to mediate resistance to a cardiotropic RNA virus, flock house virus (40). The effects of K^+^ inhibition on BUNV infection were therefore assessed in C6/36 (*Ae. albopictus* cells) as a representative cell line for the BUNV arthropod host (41). BUNV-N production was highly sensitive to K^+^ channel blockade as TEA ≥2 mm and KCl ≥20 mm inhibited BUNV-N production to almost undetectable levels (Fig. 2*C*). These data confirmed that BUNV infection of both mammalian and mosquito cells is similarly inhibited by K^+^ channel modulation.

##### K^+^ Channel Dependence Is Bunyavirus Specific

**FIGURE 3.**
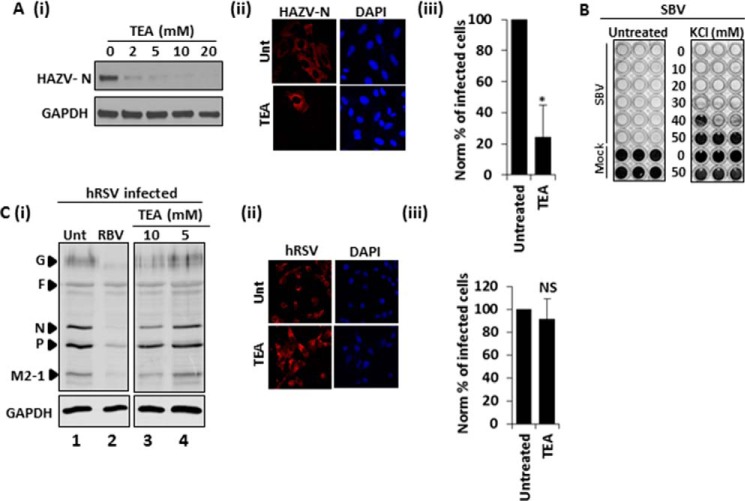
**K^+^ channel dependence is bunyavirus specific.** *A*, A549 cells were infected with HAZV (24 h, MOI 1) in the presence of the indicated concentrations of TEA. Lysates were resolved by SDS-PAGE and probed with sheep anti-HAZV N serum. Blots are representative of two independent experiments. (ii) Cells were assessed for HAZV infection ± TEA (10 mm) by staining fixed cells with sheep anti-HAZV serum and sheep Alexa fluor 594 antibodies. Representative confocal images are shown. (iii) The % of HAZV-infected cells was assessed ± TEA treatment from widefield images of HAZV-N stained cells. A minimum of 40 cells were counted. Results are expressed as mean ± S.E. (*, significant difference at the *p* ≤ 0.05 level; *NS*, not significant). *B*, Vero cells were infected with TCID_50_/ml SBV in the presence of KCl for 5 days. Cells were fixed in 100% ethanol and stained with 3% (*w*/*v*) crystal violet. Representative plates of three independent experiments are shown. *C* (i) A549 cells were infected with hRSV (24 h, MOI 1) ± the indicated concentrations of TEA or ribavirin. Lysates were resolved by SDS-PAGE and probed with goat anti-hRSV antibodies (*n* ≥ 3). Blots were reprobed for GAPDH as a loading control. Lanes shown are from the same gel and representative of three independent experiments. (ii) A549 cells were infected with hRSV ± 10 mm TEA and the % of hRSV-infected cells assessed by immunofluorescence through staining with goat anti-hRSV antibodies and anti-goat Alexa fluor 594 antibodies. Representative confocal images are shown. (iii) Cells treated as in (ii) were assessed for the % of infected cells by FACs analysis. Results are the average of two independent experiments. Values are normalized to infected no-drug controls. Results are expressed as mean ± S.E. (*, significant difference at the *p* ≤ 0.05 level; *NS*, not significant).

The diversity within bunyaviruses is revealed by their division into five distinct genera. To assess whether growth of other bunyaviruses exhibited similar dependence on K^+^ channel activity, Hazara virus (HAZV); a member of the *Nairovirus* genus, and a close relative of the highly pathogenic hazard group 4 virus Crimean Congo hemorrhagic fever virus (CCHFV; Ref. 42) was assessed. HAZV was similarly inhibited in the presence of TEA ≥ 2 mm, as judged by a clear decrease in the abundance of HAZV-N protein and the reduction of the number of HAZV infected cells treated with TEA, (Fig. 3*A*, 59.97% reduction ± 20.37 *p* ≤ 0.05). Production of the orthobunyavirus SBV was also sensitive to K^+^ channel inhibition through KCl treatment. Treatment of SBV-infected Vero cells with ≥30 mm KCl abrogated SBV-induced CPE in a manner identical to BUNV (Fig. 3*B*). To investigate if K^+^ channel sensitivity occurs during the replicative cycle of other unrelated negative sense RNA virus families, human respiratory syncytial virus (hRSV), a non-segmented RNA virus of the family *Paramyxoviridae* was assessed under identical conditions of TEA treatment. Unlike ribavirin, which was strongly inhibitory to hRSV production as expected from previous work (43), A549 cells treated with TEA concentrations of 5 or 10 mm could support hRSV infection as evidenced by hRSV protein production and the comparable numbers of hRSV infected cells compared with no-drug controls, (Fig. 3*C*, *p* ≥ 0.05). The fact that hRSV infection levels were identical to untreated controls in the face of K^+^ modulation ruled out any effects of K^+^ channel inhibition on general cell/virus metabolic processes. Similarly we have previously reported that both TEA and KCl do not impact hepatitis C virus replication at these concentrations in Huh7 cells, which were permissive to BUNV infection (Fig. 2), (22). Taken together, these data indicate a specific process during the bunyavirus lifecycle is impeded by K^+^ channel inhibition.

##### K^+^ Channel Function Is Required at an Early Stage of BUNV Infection

**FIGURE 4.**
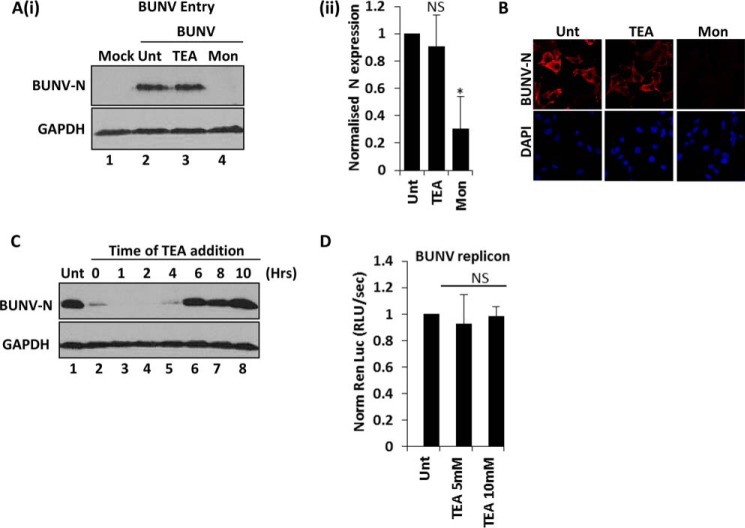
**K^+^ channel activity is required at an early post-entry stage of BUNV infection.** *A*(i), cells were infected with BUNV in the presence of TEA (10 mm) or monensin (20 μm) for 1 h. Cells were washed with PBS + 0.5% trypsin to remove cell attached viruses and cells incubated for a further 24 h in medium alone. BUNV-N expression as a marker of BUNV infection was assessed by Western blot analysis (i), densitometry (ii), and immunofluorescence (*B*). *C*, TEA was added at the indicated time points post-BUNV infection and BUNV-N expression assessed by Western blot analysis 24 h post-infection. *Unt* (untreated) is no drug included during the time course, T = x indicates the inclusion of TEA throughout the time course of BUNV infection (*n* = 3). *D*, to assess BUNV RNA synthesis, BSRT7 cells were transfected with cDNA plasmids encoding BUNV S and L segment ORFs, and a model negative sense RNA segment that contained conserved BUNV 3′ and 5′ non-translated regions flanking the *Renilla* luciferase ORF. Cells were incubated with or without 10 mm TEA, and 18 h post transfection cell lysates were analyzed for luciferase activity (RLU per second) as a measure of BUNV gene expression. The L ORF plasmid was omitted to act as a negative control to measure non-BUNV mediated luciferase expression. Results were calculated relative to those for an untreated control. Error bars represent the standard errors of the means (S.E.) of results from three independent experiments performed in duplicate. *NS*, no differences at a significance level of 0.05.

The stage of the BUNV lifecycle that requires K^+^ channel activity was next investigated. To assess the earliest events of virus infection, A549 cells were incubated with BUNV in the presence of TEA (10 mm) for 1 h at 37 °C. Virus/drug was then removed and the cells incubated for a further 24 h to allow infection to proceed (Fig. 4*A*, *i* and *ii*) respectively). Monensin (20 μm), an inhibitor of clathrin-mediated endocytosis (CME), was included in the 1-h treatment as a positive control (44). Under these conditions, TEA had no impact on BUNV attachment/entry compared with the untreated cells (Fig. 4*A* (*i*) *lanes 2* and *3*, *p* ≥ 0.05). In contrast, CME inhibition with monensin significantly impeded BUNV entry (Fig. 4*A* (*i-ii*), 60.27% ± 20.23 reduction in BUNV-N, *p* ≤ 0.05). This strongly suggested that K^+^ channel inhibition does not inhibit BUNV infection at the stage of virus entry. To observe later lifecycle events, time-of-addition experiments were performed. Cells were infected for 60 min and washed, and incubation continued at 37 °C for 24 h in the presence of TEA added at defined timepoints post-infection. When TEA was added 1–2 h post infection (hpi), BUNV-N expression was not detected (Fig. 4*B*, compare *lanes 1* and *2–4*). BUNV-N expression began to recover when treatment was delayed to 4 hpi, and was unaffected when treatment was initiated at 6 hpi. Thus, BUNV replication is restricted by synchronous or early treatment with K^+^ channel blockers (≤ 4 hpi) but not by treatment at later timepoints when virus gene expression has been initiated (≥ 6 hpi). From the observed kinetics of virus inhibition it was not clear whether the virus was inhibited at a stage of entry/uncoating or the initial steps of virus replication. The effects of TEA on BUNV-specific mRNA transcription and RNA replication (events that take place following uncoating of BUNV in the cytoplasm and subsequent release of the ribonucleoprotein (RNP) particles) were assessed using artificial BUNV minigenome assays, containing *Renilla* luciferase as a reporter (45). This approach allowed the assessment of BUNV-specific RNA synthesis independently from virus entry stages, as all components of the replicon assay are delivered by plasmid transfection, thus circumventing initial stages of the BUNV lifecycle. TEA did not affect these processes with similar levels of BUNV transcription observed between untreated *versus* TEA (5 or 10 mm) treated cells (Fig. 4*D*, *p* ≥ 0.05). This confirmed that the K^+^ channel-dependent effect during BUNV infection occurs at a post-entry stage that is prior to viral RNA synthesis.

##### BUNV Enhances Cellular K^+^ Channel Activity

**FIGURE 5.**
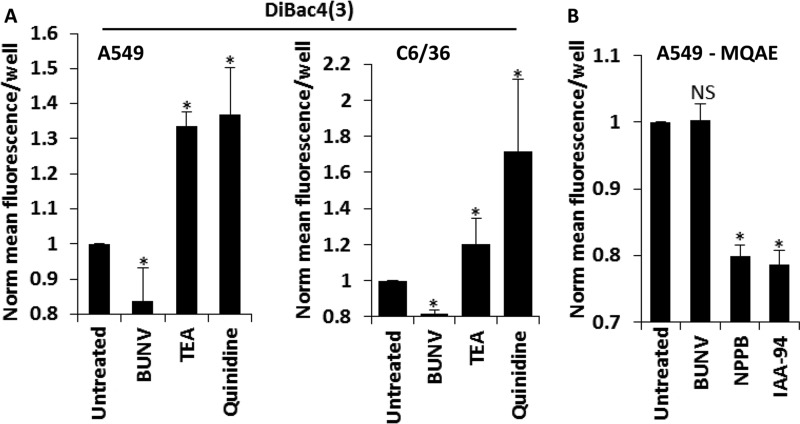
**BUNV hyperpolarises the membrane potential through K^+^ channel activation.** *A* (i) A549 or (ii) C6/36 cells infected with BUNV (MOI 1), or treated with TEA (10 mm)/quinidine (200 μm) were exposed to the membrane potential sensitive fluorescent dye DiBAC4 (3) (20 μm). Values represent 6 hpi/treatment and are normalized to untreated controls (*n* = 3). Results are expressed as the mean fluorescence per well ± S.E. of averaged widefield images (*, significant difference from untreated cells at the *p* ≤ 0.05 level). Data are representative of at least three independent experiments. *B*, A549 cells infected with BUNV (MOI 1) or treated with NPPB (10 μm)/IAA-94 (100 μm) were exposed to MQAE. Fluorescence was acquired as in Fig. 5*A*. Values represent 6 hpi/treatment and are normalized to untreated controls (*n* = 3). Results are expressed as the mean fluorescence per well ± S.E. of averaged widefield images (*, significant difference from untreated cells at the *p* ≤ 0.05 level). Data are representative of three independent experiments.

As BUNV is dependent on K^+^ channel activity during the early stages of the virus lifecycle, its ability to modulate the functionality of these channels was assessed. K^+^ channels play a crucial role in setting the resting membrane potential (the charge difference across the cell membrane). The membrane potential can either become more negative (hyperpolarization) or more positive (depolarization) compared with the resting potential as various ion channels open. In general, the activation of K^+^ channels would be expected to cause hyperpolarization that can be monitored using a membrane potential-sensitive dye, bis (1,3-dibutylbarbituric acid) trimethine oxonol; DiBAC4(3)) (46). Influx of DiBAC4(3) and an increase in fluorescence indicates cell depolarisation, while hyperpolarization is indicated by a decrease in fluorescence intensity (47). Potential-dependent fluorescence changes generated by DiBAC4(3) are typically ∼1% per mV (46). A549 cells infected with BUNV exhibited a hyperpolarised resting membrane potential 6 hpi compared with uninfected cells, as indicated by the decrease in DiBAC4(3) fluorescent intensity (Fig. 5*A*, −16.23 mV ± 14 shift). Such a change is consistent with enhanced K^+^ channel activity during the early stages of BUNV infection. To validate the assay, TEA (10 mm) and quinidine (200 μm) were included during membrane potential measurements and led to depolarization as would be expected to occur in the face of K^+^ channel blockade (Fig. 5*A*). Importantly, these effects were reproducible in C6/36 cells where BUNV infection led to a significant reduction in DiBAC4(3) fluorescence 6 hpi (Fig. 5*A*, −18.3mV ± 1.5mV shift in BUNV cells). To confirm these shifts were K^+^ channel specific, we examined Cl^−^ homeostasis during BUNV infection using the fluorescent indicator *N*-ethoxycarbonylmethyl-6-methoxy-quinolinium bromide (MQAE), a dye quenched by enhanced Cl^−^ influx and the subsequent increase in intracellular chloride concentration [Cl^−^]*_i_*. Fig. 5*B* demonstrates that BUNV did not affect [Cl^−^]_i_ compared with uninfected A549 cells (*p* ≤ 0.05), consistent with no effects on basally active Cl^−^ conductance. MQAE fluorescence was reduced by treatment with NPPB and IAA-94 (known Cl^−^ channel modulators). Taken together, these data provide evidence that BUNV specifically alters the functionality of K^+^ channels during early stages of the virus replicative cycle to regulate cellular K^+^ flux in both vertebrate and invertebrate cell lines.

##### Investigating the K^+^ channel Families Required during BUNV Infection

**FIGURE 6.**
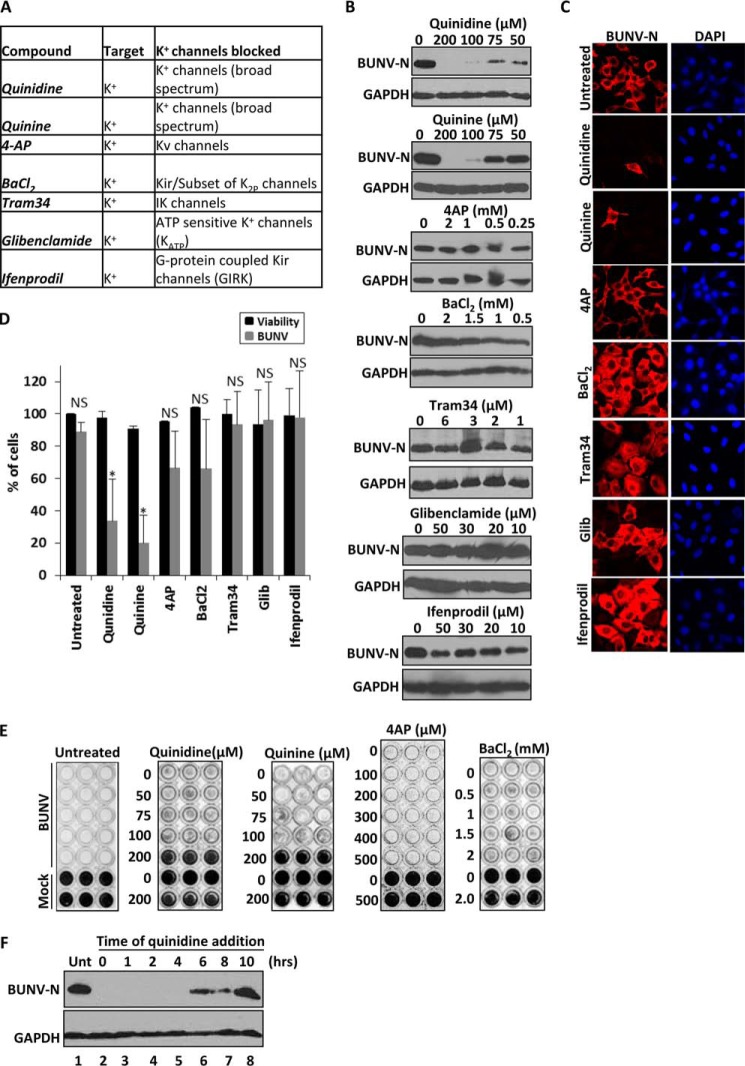
**Investigating the K^+^ channel families required during BUNV infection.** *A*, table detailing the K^+^ channel modulating compounds assessed for their effects on BUNV. *B*, BUNV-infected A549 cells (24h, MOI 1) were treated with the indicated concentrations of K^+^ channel modulators. Lysates were resolved by SDS-PAGE and probed with sheep anti-BUNV N serum. *C*, cells treated with quinidine (200 m), quinine (200 m), 4AP (1 mm), BaCl_2_ (2 mm), Tram 34 (3 m), glibenclamide (20 μm), and ifenprodil (30 μm) during the 24-h infection period were fixed, permeabilized, and stained for DAPI (*blue*) and BUNV-N (*red*) as in Fig. 1. *D*, % of BUNV infected cells was assessed for each treatment from widefield images of BUNV-N stained cells (*gray bars*). A minimum of 100 cells were counted from at least three independent experiments. ATPlite assays were performed for each drug treatment for 24 h and normalized to no-drug controls to assess cell viability (*black bars*). Results are expressed as mean ± S.E. (*, significant difference at the *p* ≤ 0.05 level). *E*, TCID_50_/ml BUNV assays were performed for selected drug treatments as in Fig. 2*D*. Representative plates of three independent experiments are shown. *F*, quinidine was added at the indicated time points post-BUNV infection and BUNV-N expression assessed by Western blot analysis 24 h post-infection. *Unt* is no drug included during the time course, T = 0 indicates the inclusion of quinidine throughout the time course of BUNV infection (*n* = 3).

**FIGURE 7.**
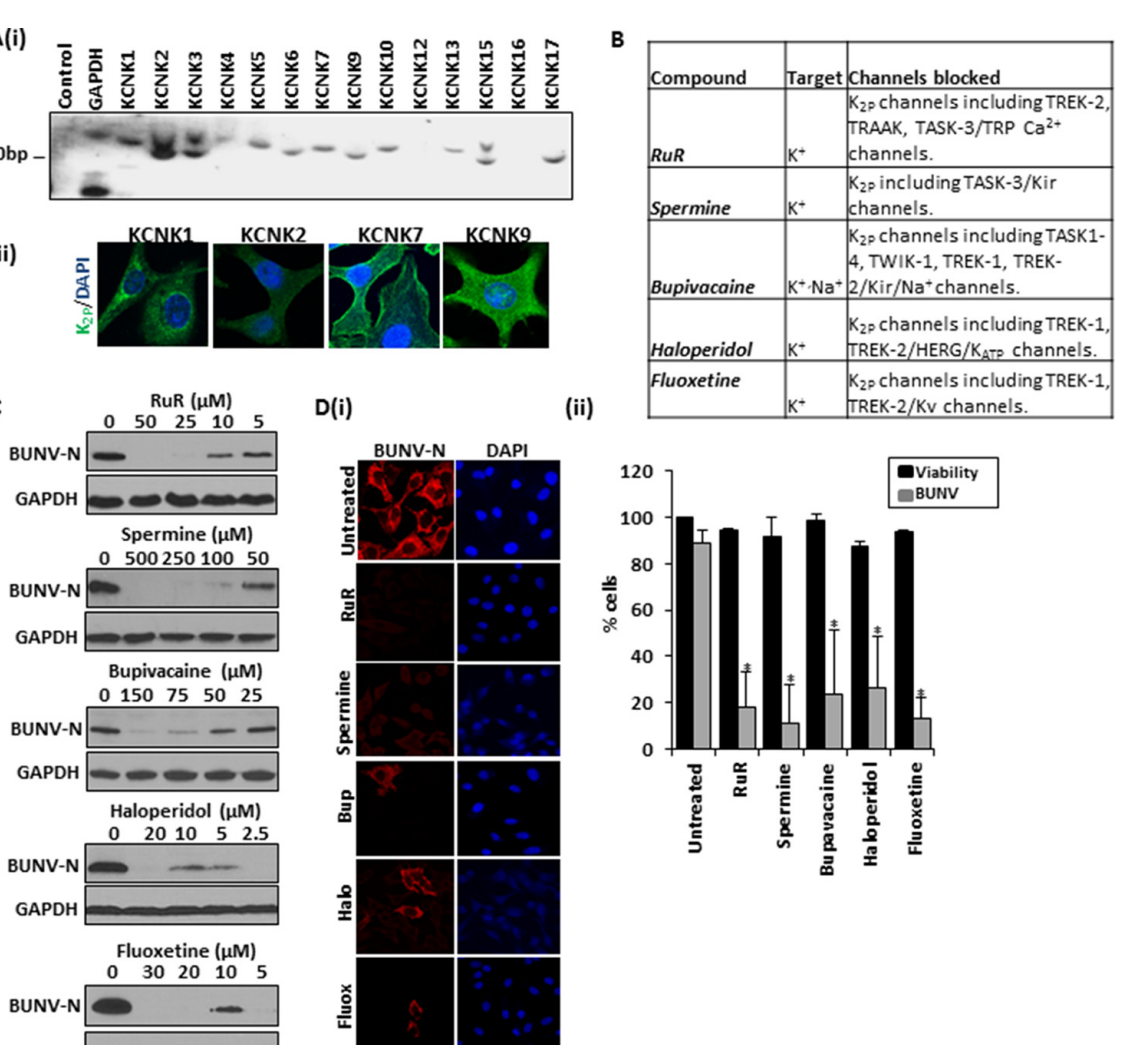
**Two-pore potassium channel blockers inhibit BUNV infection.** *A*(i), RT-PCR demonstrates that several K_2P_ channels are expressed in A549 cells. Templates for the reactions were derived from uninfected A549 cells. Negative control reactions lacked template during amplification. Identical results were obtained with at least two additional preparations of RNA. GAPDH was included as a control. (ii) Indicated K_2P_ channels were assessed for expression in A549 cells through immunofluorescent analysis. Representative confocal images are shown. *B*, table detailing the K_2P_ modulating compounds investigated during the study. *C*, BUNV-infected A549 cells (24 h, MOI 1) were treated with a range of compounds shown previously to inhibit members of the K_2P_ channel family_._ Lysates were resolved by SDS-PAGE and probed with sheep anti-BUNV-N serum. *D*(i), cells treated with RuR (50 μm), spermine (500 μm), bupavacaine (150 μm), haloperidol (20 μm), and fluoxetine (30 μm) were infected as in *B* and fixed, permeabilized, and stained for DAPI (*blue*) and BUNV-N (*red*). (ii) The % of BUNV infected cells was assessed for each treatment from widefield images of BUNV-N stained cells (*gray bars*). A minimum of 100 cells were counted from three independent experiments. ATPlite assays were performed for each drug treatment and normalized to untreated controls to assess cell viability (*black bars*). Results are expressed as mean ± S.E. (*, significant difference at the *p* ≤ 0.05 level).

Having determined that BUNV cannot establish a productive infection in the face of K^+^ channel blockade, the molecular identity of the specific K^+^ channel(s) required during the BUNV lifecycle were investigated. Over 70 different genes encode K^+^ channel subunits in the human genome, which are divided into subfamilies of voltage-gated K^+^ channels (Kv), calcium-activated K^+^ channels (BK), inwardly rectifying K^+^ channels (Kir) and two-pore K^+^ channels (K_2p_) channels (48). Quinidine and quinine block specific members of all of these subfamilies (Fig. 6*A*) and significantly inhibited BUNV-N production and reduced the number of BUNV-infected cells (quinidine ∼66.2% ± 25.7, quinine ∼79.8% ± 17.0 reduction in BUNV-infected cells, compared with untreated controls (Fig. 6, *B--D*, *p* ≤ 0.05). 4-Aminopyridine (4AP), an inhibitor of Kv channels (49), and barium chloride (BaCl_2_), an inhibitor of Kir channels (50, 51), did not impede BUNV infection suggesting that neither Kv nor Kir activity is required during the BUNV lifecycle (Fig. 6, *A--D*, *p* ≥ 0.05). Consistent with these findings, cells were protected from BUNV-induced CPE in a concentration-dependent manner by both quinine and quinidine, but virus growth was unaffected in the presence of either 4AP or BaCl_2_ (Fig. 6*E*). Similarly SBV growth was strongly inhibited by quinine and quinidine treatment but was unaffected by 4AP or BaCl_2_ (data not shown). Modulation of Ca^2+^-activated K^+^ channels (Tram 34), ATP-sensitive K^+^ channels (Glibenclamide) and G-protein-coupled Kir channels (Ifenprodil) also did not influence BUNV-N production suggesting these K^+^ channel family members do not contribute to BUNV growth (Fig. 6, *A–D*). Time-of-addition experiments for quinidine (Fig. 6*F*) additionally confirmed the drug inhibits BUNV at an identical stage to that observed for TEA (Fig. 5*C*); quinidine inhibited BUNV-N production when administered at 0 to 4 hpi and a recovery of BUNV-N production occurred when administered ≥ 6 hpi (Fig. 6*F*, compare *lanes 1*, *6–8*). Taken together, these data suggest that BUNV requires the function of quinine- and quinidine-sensitive host cell K^+^ channel(s) that are unlikely to be members of the Kv, BK, and Kir families. Given the lack of effect of Kv, BK, and Kir modulation on BUNV infection, the role of the K_2P_ channel superfamily during the BUNV lifecycle were investigated (52–54). In mammals, seventeen K_2P_ channel genes have been identified and the expression of 12 of these members (KCNK 1–7, 9, 10, 13, 15, and 17) were detected in A549 cells by RT-PCR analysis confirming their potential involvement in the BUNV lifecycle (Fig. 7*A*(i)). Protein expression was further confirmed for KCNK1, KCNK2, KCNK7, and KCNK9 by immunofluorescent staining (Fig. 7*A*(*ii*)). K_2P_ channels are distinguished by the presence of two K^+^ pores arranged in tandem, sensitivity to quinine and quinidine, and a relatively poorly defined pharmacology compared with other K^+^ family members (52, 55, 56). However, certain organic compounds have proven useful to indicate physiological roles of many K_2P_ family members (Fig. 7*B*). The organic polycations ruthenium red (RuR) and spermine can directly block specific K_2P_ channels through the interaction with negatively-charged residues in the K_2P_ pore domains (52, 57). Both compounds inhibited BUNV-N production in a concentration-dependent manner (Fig. 7*C*) and significantly reduced the percentage of BUNV-infected cells (RuR (50 μm) ∼82.1% ± 15.21 reduction, spermine (500 μm) ∼88.5% ± 16.61 reduction, *p* ≤ 0.05), compared with untreated controls (Fig. 7*D* (*i-ii*) *gray bars*) with no cytotoxicity at the highest concentration used (Fig. 7*D* (*ii*) *black bars*). In addition, drugs approved by the U.S. Food and Drug Administration (FDA), including bupivacaine (local anesthetic), haloperidol (antipyschotic), and fluoxetine (anti-depressant), similarly inhibited BUNV-N production and reduced the percentage of BUNV-infected cells at concentrations reported as inhibitory to K_2P_ channels (bupivacaine (150 μm) ∼76.8%± 27.9, haloperidol (20 μm) ∼76.3% ± 22.4 inhibition, fluoxetine (30 μm) ∼87.2%± 8.9 inhibition), (Fig. 7, *C* and *D*, *p* ≤ 0.05) (52, 56, 58). We additionally observed inhibition of BUNV by curcumin and genistein, both of which are known blockers of K_2P_ members (data not shown). These findings implicate K_2P_ channels as the K^+^ channel family member required by BUNV during the early stages of virus infection.

## Discussion

Ion channels underpin an array of essential cellular processes and drugs acting on specific ion channels are the treatment of choice for many diseases (48). Using the available pharmacological tools targeting various cellular ion channels, this study showed that K^+^ channel function is critical to the growth of BUNV (Figs. 1–2), the prototypic bunyavirus in both vertebrate and invertebrate cells. K^+^ channel dependence was also observed with HAZV and SBV, revealing this as a key virus-host interaction across other bunyaviruses (Fig. 3), suggesting it may be a general property that applies to all family members.

It is not clear why the BUNV replicative cycle is dependent upon manipulation of host cell K^+^ channel currents. Previous reliance of bunyavirus replication had been noted by Frugulhetti and Rebello (59), who reported that changes in K^+^ ion concentrations disrupted the growth of the orthobunyavirus Marituba virus. The stage of the life cycle affected was found to be mRNA translation, although the mechanism that underpinned this dependence was not pursued, and the role of cellular ion channels in this process was not investigated. In the present study, K^+^ channel modulation had no discernible effect on mRNA transcription and translation in the context of the BUNV minireplicon system (Fig. 4*C*) and so we suggest that our findings of K^+^ channel reliance are likely unrelated to these early findings. To better understand the nature of the K^+^ channel requirement, time of addition assays were performed which indicated that the K^+^ sensitive steps in the BUNV life cycle occur within the initial 6 h of virus infection, but do not include virus binding/entry (Fig. 4, *A* and *B*). It is thus most likely that K^+^ channel modulation contributes to early events within the virus life cycle including virus uncoating and/or events prior to the formation of RNA replication factories, postulated to assemble around the Golgi (60–62).

A key finding of this study is that K_2P_ channels are the K^+^ family member required for BUNV infection. BUNV was inhibited by diverse pharmacological agents that modulate K_2P_ channels (52, 57, 58, 63, 64), but no effects of K^+^ channel modulators targeting Kv, BK, and Kir channels was observed. The family of K_2P_ channels is small containing only ∼17 members, which regulate the membrane potential of both “excitable” and “non-excitable” cells (58). K_2P_ channels respond to a diverse array of stimuli including pH, temperature and membrane stretch and their pharmacology is highly characteristic, as a result of their distinct structural features (55, 56, 65). Using a fluorescent membrane potential probe, hyperpolarization of the plasma membrane was observed in BUNV-infected cells 6 hpi (Fig. 4), a shift consistent with an enhancement of K_2P_ activity at the stage of the virus lifecycle sensitive to K^+^ channel modulation. Many viruses encode their own viroporin (reviewed in Ref. 66), which are pore forming proteins that have been documented to participate in several viral functions, including the promotion of release of virus particles, modulation of cellular vesicles, glycoprotein trafficking, and membrane permeability. As BUNV has no known viroporin, it is possible that BUNV proteins have evolved to interfere and depend on cellular K_2P_ channels to aid virus pathogenesis. While we do not understand the mechanism(s) of K_2P_ activation, our findings implicate BUNV structural proteins present in the virion to mediate these effects since viral gene expression would not have occurred at these timepoints, and thus no new proteins would be generated. Orthobunyaviruses have been shown to enter cells via clathrin mediated endocytosis (44), but events post-entry are less well understood, although virion trafficking of CCHFV has been shown to be ESCRT dependent (67). The modulation/reliance upon host cell ion K_2P_ channels at this post-entry stage is consistent with recent observations for other negative sense enveloped viruses. Post-entry trafficking of Ebola virus, a single stranded negative sense RNA virus, is dependent upon endosomal calcium channels termed two-pore channels (TPCs) (26). In addition the capsid (C) protein of dengue virus, a single-stranded positive sense RNA virus, forms an association with lipid membranes that is intrinsically dependent on the intracellular concentrations of K^+^ ions (68). The cellular mechanisms of these ion-channel dependent events remain to be elucidated.

As HAZV represents a model for CCHFV (41), a cause of a severe, often fatal disease in humans, this study highlights the potential of K^+^ channel modulating drugs as a novel therapeutic intervention against more dangerous pathogens of this family. It is interesting to note that compounds such as haloperidol and fluoxetine possess highly desirable drug properties in a clinical setting; good oral bioavailability (60–70%), generally well tolerated with severe side effects uncommon and the ability to cross the blood brain barrier (63, 69). This may be pertinent for bunyavirus treatment since BUNV infection in both mouse and horses is largely neurotropic (70, 71). Indeed BUNV infection was permissive in U87-MG cells; a human primary glioblastoma cell line, and could be inhibited by K^+^ channel modulation (Fig. 2).

In conclusion, despite encompassing a large number of viruses capable of causing outbreaks that would impact public health, national economies and food security; little is known regarding key stages in the life cycle of bunyaviruses. This study has demonstrated the importance of K^+^ channel activity during the BUNV lifecycle and contributes to the growing field of viral ion channel interactions. Targeting these K^+^ channels in patients infected with severe *Bunyaviridae* infections may therefore represent a new prospect for future anti-viral drug development.

## Author Contributions

S. H., B. K., B. H., E. L., and H. T. performed the experiments. S. H., B. K., A. K., J. N. B., J. M. conceived the experiments. J. D., M. D., C. P., E. S., C. M., A. K. provided reagents and expertise. B. K., J. N. B., and J. M. wrote the manuscript.

## Footnotes

3 The abbreviations used are:

BUNV: Bunyamwera virus
ORF: open reading frame
RLU: relative luminescence unit
MQAE: 6-methoxy-quinolyl acetoethyl ester
NPPB: 5-nitro-1–3-phenylpropylamino benzoic acid
IAA: indyanyloxyacetic acid
TEA: tetraethylammonium
CPE: cytopathic effect
CME: clathrin-mediated endocytosis
SBV: Schmallenberg virus.
